# Shifts on Gut Microbiota Associated to Mediterranean Diet Adherence and Specific Dietary Intakes on General Adult Population

**DOI:** 10.3389/fmicb.2018.00890

**Published:** 2018-05-07

**Authors:** Izaskun Garcia-Mantrana, Marta Selma-Royo, Cristina Alcantara, María C. Collado

**Affiliations:** Institute of Agrochemistry and Food Technology, Spanish National Research Council, Valencia, Spain

**Keywords:** microbiota, feces, mediterranean diet, nutrition, diet patterns

## Abstract

There is increasing evidence for the interaction between gut microbiome, diet, and health. It is known that dysbiosis is related to disease and that most of the times this imbalances in gut microbial populations can be promoted through diet. Western dietary habits, which are characterized by high intakes of calories, animal proteins, saturated fats, and simple sugars have been linked with higher risk of obesity, diabetes, cancer, and cardiovascular disease. However, little is known about the impact of dietary patterns, dietary components, and nutrients on gut microbiota in healthy people. The aim of our study is to determine the effect of nutrient compounds as well as adherence to a dietary pattern, as the Mediterranean diet (MD) on the gut microbiome of healthy adults. Consequently, gut microbiota composition in healthy individuals, may be used as a potential biomarker to identify nutritional habits as well as risk of disease related to these habits. Dietary information from healthy volunteers (*n* = 27) was recorded using the Food Frequency Questionnaire. Adherence to the MD was measured using the PREDIMED test. Microbiota composition and diversity were obtained by 16S rRNA gene sequencing and specific quantitative polymerase chain reaction. Microbial metabolic activity was determined by quantification of short chain fatty acids (SCFA) on high performance liquid chromatography (HPLC). The results indicated that a higher ratio of Firmicutes–Bacteroidetes was related to lower adherence to the MD, and greater presence of Bacteroidetes was associated with lower animal protein intake. High consumption of animal protein, saturated fats, and sugars affected gut microbiota diversity. A significant higher presence of Christensenellaceae was found in normal-weight individuals compared to those who were overweight. This was also the case in volunteers with greater adherence to the MD compared to those with lower adherence. *Butyricimonas*, *Desulfovibrio*, and *Oscillospira* genera were associated with a BMI <25 and the genus *Catenibacterium* with a higher PREDIMED score. Higher bifidobacterial counts, and higher total SCFA were related to greater consumption of plant-based nutrients, such as vegetable proteins and polysaccharides. Better adherence to the MD was associated with significantly higher levels of total SCFA. Consequently, diet and specific dietary components could affect microbiota composition, diversity, and activity, which may have an effect on host metabolism by increasing the risk of Western diseases.

## Introduction

A structured and equilibrated gut microbiome is needed for optimal health status. Factors such as antibiotic use, cesarean-section deliveries, excessive hygiene, stress, and lack of exercise have a major impact on the microbiome (Penders et al., 2006; Dominguez-Bello et al., 2010; Rodríguez et al., 2015). Diet is considered one of the major drivers for gut microbiota composition (Graf et al., 2015). A rich and balanced diet is essential to promote the maintenance of diversity and proper functioning of a healthy gut microbiota (Heiman and Greenway, 2016). Nowadays, modern societies are exposed to a “Western lifestyle,” which is characterized by an excessive intake of energy-dense foods rich in fats, cholesterol, animal proteins, sugars, salt and a wide range of processed foods, along with lack of exercise that promote an inflammatory state (Myles, 2014). This low grade inflammation may trigger the development of several disorders, such as obesity, metabolic syndrome, cardiovascular diseases, and colorectal cancer (Minihane et al., 2015). In addition to diet, exercise is another important environmental factor that can influence the gut microbiota composition with possible benefits for human health (Monda et al., 2017). The prevalence of obesity is increasing and today more than half of the world’s population is considered to be overweight. We have to take into account that an overweight status is the initial step for obesity development. Overweight is very common in general population. Although many of them are considered “healthy population” if they don’t present any disorder or healthy problem, they are more prone to develop a disease later in life (Biro and Wien, 2010). Nowerdays, most of this human disorders have been associated with alterations in microbiota composition and at same time, a reduced bacterial richness and diversity (Cani et al., 2009; Le Chatelier et al., 2013). Although there is increasing evidence of the influence of several nutrients on gut microbiota composition, the effect on microbiota of a global dietary pattern such as the MD has not been sufficiently studied although there is evidence on beneficial influence of MD on health. It is well assessed the role of MD in preventing non-communicable disorders, as cardiovascular diseases, type 2 diabetes, obesity and cancer (Sofi et al., 2013). The MD has a greater emphasis on vegetables, fruits and pulses, which are also associated with reduced risk of developing Western diseases (Ogce et al., 2008). Due to bacterial fermentation of complex carbohydrates present in these groups of foods, a healthy microbiota produces large quantities of biologically active short-chain fatty acids, such as acetate, butyrate and propionate. These positively influence health status (Rivière et al., 2016). Furthermore, de Filippis and colleagues also reported that high level consumption of plant based foods and high-level adherence to a MD beneficially impacts the gut microbiota and associated metabolomic profile (De Filippis et al., 2016). Despite the relevance of this type of diet to a healthy microbiota, there is little information about the impact of diet and specific dietary components on intestinal microbiota in adults without associated pathology. Feeding pattern and dietary composition are important parameters when assessing the microbiome’s contribution to human metabolism. However, too little is known about the effect of diet and dietary compounds on the microbiota. This study aimed to fill this gap in knowledge by determining the effect of food consumption and adherence to the MD on the gut microbiome of normal adults without pathology.

## Materials and Methods

### Subjects and Sampling

A total of 27 volunteers (16 females and 11 males) ranged 39.5 ± 7.3 years old and living in a Mediterranean area (Valencia-SPAIN) participated in this study. Clinical, anthropometric and nutritional characteristics were recorded (**Table 1**). None of the volunteers recruited for the study had non-declared pathology, neither had been treated with antibiotics, probiotics or prebiotics at least within the last 2 months before the study started. Height and weight was measured and BMI was calculated by: weight (kg)/height (m2) and stratified according to Sociedad Española para el Estudio De la Obesidad (SEEDO) criteria (Salas-Salvadó et al., 2007) :lean-normal 18.5–25.0 Kg/m2), over-weight (25.1–29.9 kg/m2), and obese (≥30.0 kg/m2). Volunteers were given oral and written instructions for the standardized collection of fecal samples. Feces were collected by each volunteer at home using fecal plastic recipients and then, those were placed in the freezer at -20°C overnight, before being send to the Institute of Agrochemistry and Food Technology, where the samples were stored at -80°C until analysis.

**Table 1:** Clinical and nutritional characteristics of the participants.

	Age (years) (mean ± SD)	BMI kg/cm2 (mean ± SD)	Mediterranean diet (Prevalence)^∗^
Male	38.72 ± 6.11	25.29 ± 2.76	(5/11)
Female	40.01 ± 7.92	21.95 ± 2.72	(10/16)


*^∗^Prevalence = number of individuals from total number of volunteers with PREDIMED score higher than 9 (adherence to Mediterranean diet).*

Written informed consent was obtained from all volunteers, and the study protocol was approved by the local ethics committee of the Atencion Primaria-Generalitat Valenciana (CEIC-APCV). All protocols and methods were performed in accordance with the relevant guidelines and regulations.

### Dietary Estimation

To determine dietary intake of the previous year each participant was asked to answer a full-length 140-item validated FFQ food frequency questionnaire (Fernández-Ballart et al., 2010). In any case, we also validated the FFQ with a 3-day food records questionnaire for the intake of dietary nutrients. They were analyzed by EASY DIET program that use the nutrient Food Composition Tables developed by the Centro de Enseñanza Superior de Nutrición Humana y Dietética (CESNID). We obtained daily intakes estimates about total energy intake, macro, micronutrients and total dietary fiber. Data were normalyzed by energy intake. As cutoff we used the media of dietary nutrients. We establish that values above the media were considered as a high intake whereas values below the media were considered as low intake. A 14-item questionnaire, PREDIMED validated test, was also used in this study to appraise adherence of participants to the MD (Martinez-Gonzalez et al., 2012). The MD score ranged from 0 (minimal adherence) to 14 (maximal adherence). As cutoff point it was used a score of nine points. A score of nine or more means good adherence to the MD.

### Short Chain Fatty Acids (SCFA) Analysis

An aliquot of 100 mg of fecal sample was diluted in 1 ml of phosphoric acid 0.1% as described elsewhere (Sarmiento-Rubiano et al., 2007). Samples were vortexed and then centrifuged at 13000 × *g* for 5 min at 4°C. Supernatants were filtered through 0.45 μm-pore-size nitrocellulose filters (Millipore, United States) and stored at -80°C until HPLC analysis. SCFAs analysis was performed by using high pressure liquid chromatographic methods (Jasco Corporation, Japan). 20 μl of each sample was injected into the HPLC system, that comprised a column Rezex^TM^ ROA-Organic Acid H+ (8%), LC Column 300 × 7.8 mm, (Phenomenex, United States), that was placed at 30°C and a UV detector at 210 nm. Phosphoric acid 0.1% was used as a mobile phase at a flow rate of 0.5 ml/min. Standards curves for formic, lactic, acetic, propionic, isobutyric, butyric, isovaleric, and valeric acid were used for quantify the SCFAs in fecal samples. SCFAs concentrations were expressed in mM.

### DNA Extraction

Total DNA was extracted from the fecal material (approx. 100–200 mg) using the Master-Pure DNA extraction Kit (Epicentre, Madison, WI, United States) following the manufacturer’s instructions with the following modifications: samples were treated with lysozyme (20 mg/mL) and mutanolysin (5 U/mL) for 60 min at 37°C and a preliminary step of cell disruption with 3-μm diameter glass beads followed by 1 min at 2,000 oscillations by bead beater. Purification of the DNA was performed using DNA Purification Kit (Macherey-Nagel, Duren, Germany) according to manufacturer’s instructions. DNA concentration was measured using Qubit^®^ 2.0 Fluorometer (Life Technology, Carlsbad, CA, United States) for further analysis.

### Bacterial Profile Quantification by Quantitative PCR Analysis (qPCR)

Quantitative polymerase chain reaction was targeted to quantify total bacteria, Enterobacteriaceae family, *Bifidobacterium* group, *Bacteroides-Prevotella-Porphyromonas* group, *Bacteroides fragilis* group, *Blautia coccoides* group, *Methanobrevibacter smithii, Faecalibacterium prausnitzii* as described previously (Collado et al., 2008; Mira-Pascual et al., 2015). The qPCR amplification and detection were performed by duplicate on a LightCycler 480 Real-Time PCR System (Roche Technologies). Each reaction mixture of 10 mL was composed of SYBR Green PCR Master Mix (Roche), 0.5 mL of each primer (concentration 10 mmol/L), and 1 mL of DNA template. The bacterial concentration in each sample was calculated by comparison with the Ct values obtained from standard curves that were obtained by serial tenfold dilution of specific DNA fragments.

### Microbiota Profiling and Bioinformatics Analyses

The V3-V4 variable region of the 16S rRNA gene was amplified by PCR using Illumina adapter overhang nucleotide sequences, following Illumina protocols. After 16S rDNA gene amplification, the multiplexing step was performed using Nextera XT Index Kit (Illumina, San Diego, CA, United States). 1 μl of the PCR product was checked with a Bioanalyzer DNA 1000 chip (Agilent Technologies, Santa Clara, CA, United States) and libraries were sequenced using a 2 × 300 pb paired-end run (MiSeq Reagent kit v3) on a MiSeq-Illumina platform (FISABIO sequencing service, Valencia, Spain) according to manufacturer’s instructions (Illumina). After Quality assessment, sequence joining and chimera removal as described (Rodríguez-Díaz et al., 2017), sequences were then binned into Operational Taxonomic Units (OTUs) using an open reference OTU picking method using 97% identity to the Greengenes 13_8 database using the QIIME pipeline (version 1.9.0) (Caporaso et al., 2010). Samples were rarefied to 12.111 reads per sample, and then evaluate for diversity. OTUs with a relative frequency below 0.01 were removed. Sequences that could not be classified to domain level, or were classified as Cyanobacteria and Chloroplasts, were removed from the dataset as they likely represent ingested plant material. Alpha diversity (Chao1 and Shannon indexes) and beta diversity were also determined. Calypso software version 8.00^1^ was used with total sum normalization for the statistical analysis, multivariante test and data mining. Data were classified by metadata factors and differences in relative abundance were evaluated using *T*-test. Linear discriminant analysis (LDA) effect size (LEfSe) algorithm (Segata et al., 2011) was used to identify specific bacterial features between conditions and diet patterns. Pearson’s correlations heatmaps between nutrients and microbial community composition were also plotted using Calypso software. Data were considered statistically significant at *P* < 0.05.

## Results

### Subjects and Sampling

Most of the volunteers (40.7%) showed good adherence to MD as high score in PREDIMED test was obtained (>9 score). Significant higher intake of dietary fiber (*P* = 0.025) was observed in the higher MD-score group compared to those observed in the lower MD score group. No significant differences in the rest of specific dietary intakes studies between MD score groups were found.

### Short Chain Organic Fatty Acids (SCFA) Analysis

**FIGURE 1:**
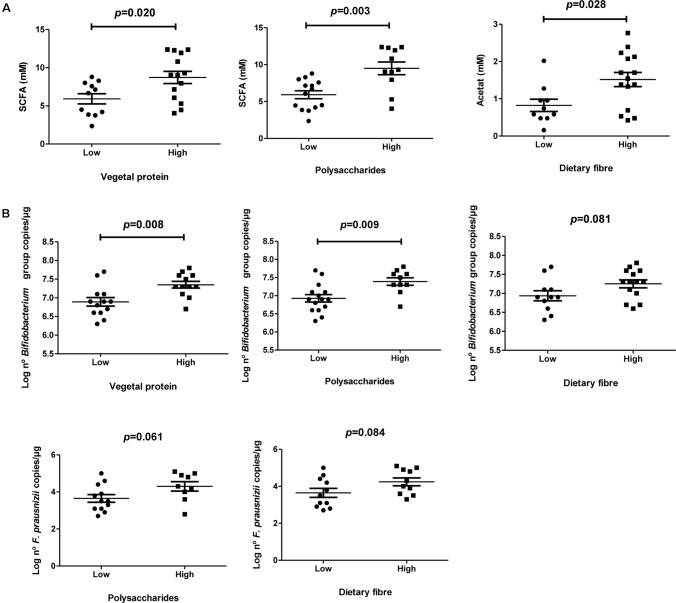
Microbiota composition and SCFA profile according to specific nutrient intake. **(A)** Total SCFA levels measured by HPLC according to specific nutrient intake as vegetal protein, polysaccharides and dietary fiber; **(B)** Specific microbial levels measured with qPCR according to specific nutrient intakes as vegetal protein, polysaccharides and dietary fiber.

No significant differences were found according to BMI. Participants who showed a better adherence to the MD are correlated with a higher total concentration of acetate, propionate, and butyrate in fecal samples (*P* = 0.023; *R* = 0.452). Higher concentrations of acetate (*P* = 0.006; *P* = 0.001), propionate (*P* = 0.016; *P* = 0.004), and total SCFA (*P* = 0.020; *P* = 0.003; **Figure 1A**) were detected in participants whose diets were higher in vegetal proteins and polysaccharides, respectively. Furthermore, a positive association was found for acetate and dietetic fiber (*P* = 0.028; **Figure 1A**).

### Bacterial Profile Quantification by qPCR Analysis

#### Bacteria Profile According to BMI and Mediterranean Diet (MD) Stratification

No significant associations between BMI or MD stratification and bacterial groups were found.

#### Bacteria Profile According to Dietary Nutrient Intake

Significant higher levels of *Bifidobacterium* spp. (**Figure 1B**) were found in individuals who consume more vegetal proteins (*P* = 0.008), carbohydrates (*P* = 0.003) and polysaccharides (*P* = 0.009) compared to those ones with lower intakes. Positive trends between dietetic fiber intake and specific levels of *Bifidobacterium* genus (*P* = 0.081) and *F. prausnitzii* (*P* = 0.084) were found. Same trend was also observed for polysaccharides and levels of *F. prausnitzii* (*P* = 0.061).

### Microbiota Composition by 16S rDNA Sequencing

**FIGURE 2:**
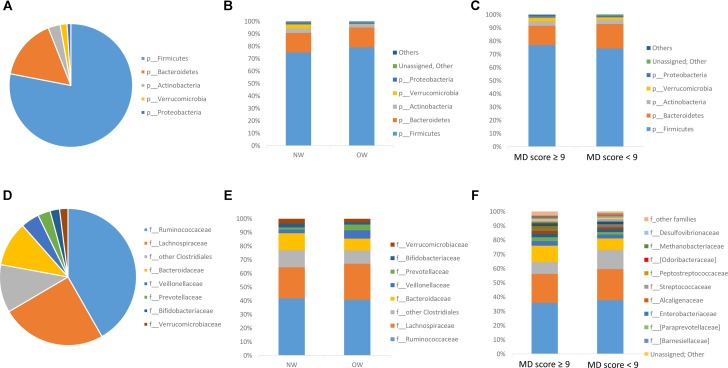
Microbiota composition by 16S rDNA sequencing. Microbial Relative abundances (%) at phylum (**A**: General profile, **B**: according to BMI, and **C**: according to MD adherence) and family level (**D**: General profile, **E**: according to BMI, and **F**: according to MD adherence) found in the gut microbiome of volunteers.

The most predominant group at phylum level (**Figure 2A**) was Firmicutes (77.31% ± 2.88) followed by Bacteroidetes (15.86% ± 0.28), Actinobacteria (3.13% ± 0.65) and Verrucomicrobia (1.78% ± 1.22) and Proteobacteria was almost 1%. At family level (**Figure 2D**), the most predominant bacterial population belonged to the Ruminococcaceae (37.97% ± 1.06), Lachnospiraceae (21.78% ± 0.71), Bacteroidaceae (13.05% ± 3.54), followed by other Clostridiales (8.09% ± 2.55), Veillonellaceae (2.67% ± 0.50) and Verrucomicrobiaceae (1.82% ± 0.63).

#### Microbiota Composition According to BMI Stratification

**FIGURE 3:**
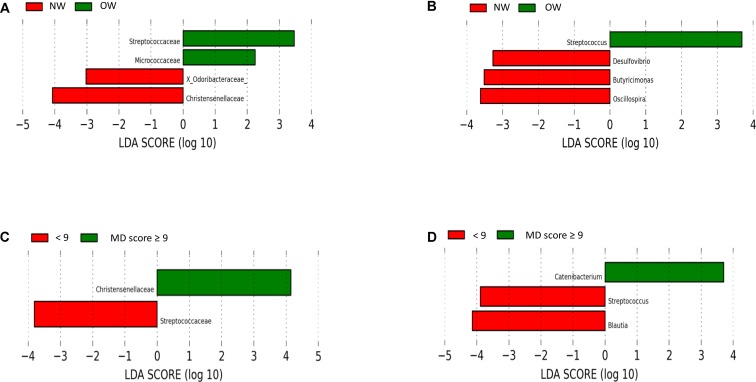
Linear Discriminant Analysis (LDA) Effect Size (LEfSe) plot of taxonomic biomarkers identified in the gut microbiome of volunteers. Specific bacterial traits found at family and genus levels according to BMI (**A,B**, respectively) and associated with the adherence to Mediterranean diet (**C,D**, respectively). The LEfSe algorithm, emphasizing both statistical and biological relevance, was used for biomarker discovery. The threshold for the logarithmic discriminant analysis (LDA) score was 3.

The most predominant groups at phylum and family level according to BMI stratification are shown in **Figure 2B** and **Figure 2E** respectively. Significantly higher levels were found between normal weight participants for Verrucomicrobia phylum compared to overweight ones. According to Lefse analysis Christensenellaceae was significantly enriched in the normal weight group and Streptococcaceae was associated with those individuals who presented a higher BMI (**Figure 3A**). In addition, at genus level, *Desulfovibrio, Butyricimonas* and *Oscillospira* was related to the normalweight group (**Figure 3B**). Alpha diversity (Shannon index) did not show significant differences in microbial diversity according to BMI (*P* = 0.105). A redundancy analysis (RDA) on the OTUs level was performed and no significant differences were found in the microbial populations according to the BMI, only a negative trend could be observed (*P* = 0.076).

#### Microbiota Composition According to MD-Score Stratification

The most predominant groups at phylum and family level according to MD-score stratification are shown in **Figure 2C** and **Figure 2F** respectively. Higher MD score was related to lower Firmicutes/Bacteroidetes ratio (*R* = -0.369; *P* = 0.057). Lefse analysis showed a higher association of Christensenellaceae with individuals with a higher MD score and of Streptococcaceae with those individuals who presented a lower adherence to the MD (**Figure 3C**). At genus level we also found significant differences. *Catenibacterium* was related to the MD group (**Figure 3D**). ANOVA test showed lower relative abundances of *Clostridium* genus for the MD group and at species level, we found higher levels of *Bacteroides uniformis* and *B. ovatus* for the participants who did not follow the MD. Alpha diversity (Shannon index) did not show significant differences according to the MD score. However, according to Chao index, gut microbial richness tended to be higher in individuals who presented a better adherence to the MD (*P* = 0.061). The RDA on the OTUs level did not show significant differences according to the MD score (*P* = 0.321).

#### Microbiota Composition According to Dietary Nutrient Intakes

**FIGURE 4:**
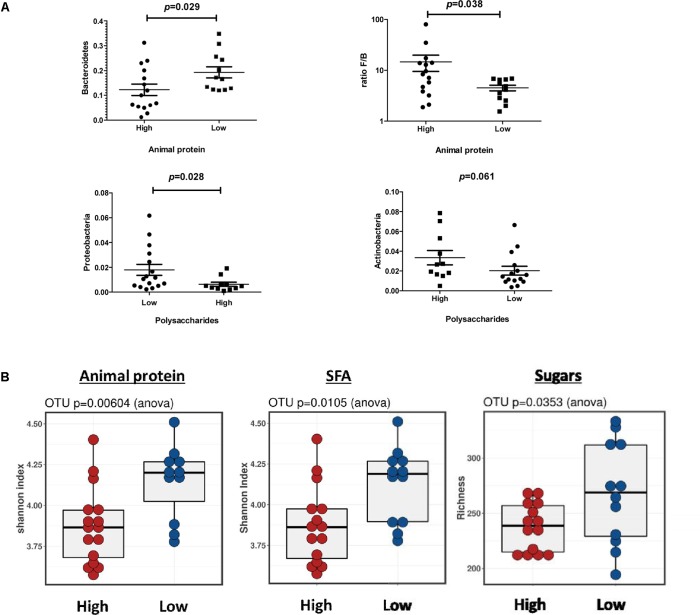
Microbiota profile and SCFA profile according to specific nutrient intake. **(A)** Significantly different taxa at phylum level related to nutrient intake. **(B)** Bacterial diversity and richness at OTUs level according to nutrient intake.

At phylum level (**Figure 4A**), higher intake of animal protein was related to a significant lower presence of Bacteroidetes (*P* = 0.029) and a higher Firmicutes/Bacteroidetes ratio (*P* = 0.038). A lower intake of polysaccharides was related to a higher relative abundance of Proteobacteria (*P* = 0.028) whereas the relative abundances of Actinobacteria tended to be lower (*P* = 0.061). At genus level, lower relative abundance of *Parabacteroides* (*P* < 0.043) and *Butyricimonas* (*P* < 0.015) was related to higher intake of animal proteins and saturated fats. Lower levels of *Butyricimonas* (*P* < 0.020) was also associated with simple sugars. Lower relative abundance of *Oscillospira* (*P* < 0.021) was found for high protein, cholesterol and total carbohydrate intake, whereas higher abundances of *Coprococcus* (*P* < 0.025) and *Bifidobacterium* (*P* < 0.013) was related to higher polysaccharides intake. Moreover, Lefse analysis showed similar significant associations respect to nutrient intakes (*P* < 0.05). We found an enrichment of *Butyricimonas* in the group of individuals with low protein, saturated fats, cholesterol and simple sugars and a predominance of *Oscillospira* in those individuals who consume less proteins and cholesterol. Lefse analysis also showed an association of *Roseburia* and the intake of vegetal proteins, *Haemophilus* for all type of proteins, saturated fats and carbohydrates as well as for *Coprobacillus* and *Streptococcus* with cholesterol. We analyzed the relationship of dietary fat type and gut microbiota composition. The genus *Dorea* and *Lactobacillus* were overrepresented in diets with a higher PUFA/SFA ratio. We also found higher enrichment of *Bifidobacterium* and *Lactobacillus* in fecal samples of individuals with high carbohydrate intake specifically a positive relationship of *Coprococcus* and *Bifidobacterium* with polysaccharides. A lower dietetic fiber consume was related to a predominance of *Blautia* and *Bulleidia*, whereas a high intake of dietetic fiber was associated with *Methanobrevibacter* genus. We also found significant differences at Operational Taxonomic Unit (OTU) level respect to microbial diversity (**Figure 4B**). We observed a lower microbial diversity (*P* < 0.05) in samples from individuals who ate higher proportions of animal protein and saturated fat along with lower microbial richness in samples from participants who consumed more simple sugars (*P* < 0.05). Moreover, we also found significant correlations between the nutrients and the relative abundances of specific gut bacteria at genus level (see **Supplementary Figure S1** and **Supplementary Table S1**).

## Discussion

Diet is one of the main factors in determining gut microbiota composition, diversity and activity (Graf et al., 2015). Dietary patterns are associated with different microbiota composition profiles (Wu et al., 2011). Western diets have been associated with less bacterial diversity and different microbial profiles when compared to traditional diets, such as the MD. There is increasing evidence suggesting that the traditional MD and other diets with similarly low animal protein and high vegetable and fiber intake are associated with the prevention of cardiovascular diseases, including reduced mortality risk and lower weight gain (Chierico et al., 2014; Graf et al., 2015). Despite the relevance of diet in microbiota composition and that diet, microbiota, and health are interrelated, there is little information about the impact of dietary patterns and specific components of diet on intestinal microbiota in adults with no associated pathology.

### BMI Stratification

The intestinal microbiota plays an important role in the maintenance of host health. Several studies have linked changes (dysbiosis) in the gut microbial community to metabolic disorders, such as obesity and insulin resistance (Le Chatelier et al., 2013). Many previous works have shown associations between various bacterial groups and weight status (Bajzer and Seeley, 2006; Ley et al., 2006; Clarke et al., 2012). In any case, there are some controversies about the composition of gut microbial communities in obese individuals. In our study, Verrucomicrobia phylum was significantly more abundant in the normal weight group, as previously reported (Clarke et al., 2012). As our results showed, members of the family Christensenellaceae and the genera *Desulfovibrio* and *Oscillospira* were more abundant in lean individuals. Karlsson and colleagues conducted a study on overweight children showing that the reduction of certain bacterial groups, such as *Desulfovibrio* and *Akkermansia muciniphila*-like bacteria, resulted in an increase in pathogens, such as members of gram-negative family Enterobacteriaceae identified as biomarkers in the development of obesity (Karlsson et al., 2012). Moreover, *Oscillospira* has been widely positively associated with leanness (Verdam et al., 2013; Escobar et al., 2014), in both children and adults, including twin studies (Tims et al., 2013; Goodrich et al., 2014). Bacteria species providing anti-inflammatory effects, as *F. prausnitzii*, are found in lean people, whereas those with pro-inflammatory effects were more likely to be present in obese individuals (Andoh et al., 2016). Walters and colleagues found lower levels of *Oscillospira* in patients with inflammatory diseases, such as Crohn’s and inflammatory bowel disease (IBD) (Walters et al., 2014). In addition, it has been also found a strong association involving *Oscillospira* with *Christensenella minuta*, which promoted leanness in inoculated germ-free mice (Goodrich et al., 2014).

### Mediterranean Diet (MD)-Score Stratification

There is growing evidence that the MD is beneficial to human health. Large cohort studies have demonstrated that good adherence to MD is related to reduced risk of developing several disorders, such as cardiovascular disease and diabetes (Mendez et al., 2006; Lopez-Legarrea et al., 2014). These also showed that the MD is related to reduced inflammation markers (Dontas et al., 2007; AlEssa et al., 2017). Mediterranean dietary patterns may be an important dietary tool against obesity (Newby et al., 2004; Schulz et al., 2005; Mendez et al., 2006). In this study, a higher presence of Bacteroidetes and a lower Firmicutes–Bacteroidetes ratio was found in individuals who consumed less animal protein and who had a higher MD score. A high Firmicutes–Bacteroidetes ratio has been related to several disorders, such as IBD (Man et al., 2011), type 2 diabetes (Larsen et al., 2010) and obesity (Turnbaugh et al., 2006). Nevertheless, no significant differences were found in levels of *Bacteroides* and *Prevotella*, genus included in Bacteroidetes phyla. In a cross-sectional study (Wu et al., 2011), it has been shown that people who followed a long term meat-rich diet had greater amounts of *Bacteroides* compared to those whose diets were rich in carbohydrates, who had higher counts of *Prevotella.* However, it is also known that *Bacteroides* possess hydrolytic abilities, which are involved in the degradation of insoluble polysaccharides. This makes them good gut colonizers (Jain et al., 2007), that are able to degrade a wide variety of polysaccharides. Most of the findings on the MD described here are in agreement with previous studies. A recent study reported higher proportions of Bacteroidetes in those subjects with better adherence to the MD (Gutiérrez-Díaz et al., 2016). In another work comparing the fecal microbiota of EU children with children from a rural African village, those whose diets were higher in fiber content had significantly higher levels of Bacteroidetes and a unique abundance of bacteria from the genus *Prevotella* and *Xylanibacter* (De Filippo et al., 2010). We found that lower adherence to the MD was associated to lower levels of *Catenibacterium* genus. Recent study explored differences in gut microbial composition in two groups of teenagers (Egypt vs. United States) according to their diets (Shankar et al., 2017). The guts of Egyptian adolescents were enriched with polysaccharide-degrading genera including *Catenibacterium* (Shankar et al., 2017). In contrast, in another recent work the genera *Catenibacterium* has been found in individuals with a high-fat diet, even if there was no evidence of an abnormal BMI (Shin et al., 2016). However, the biological implications of this remain unclear. Interestingly, we found a significant association between Christensenellaceae levels and better MD adherence as well as lower BMI. As previously described, Christensenellaceae has been associated with lean subjects (Goodrich et al., 2014) as it has been found in higher abundance in the gut microbiome of lean people compared to those of obese people. Another study (Papa et al., 2012) reported lower levels of Christensenellaceae in fecal samples of pediatric and young adult IBD patients compared with those of healthy controls. However, until now no relationship has been found between Christensenellaceae and human diet. This is an important finding of this study, because in addition to find an association of Christensenellaceae with lean individuals, we could also detect this association in those individuals who presented a better dietary pattern. In support of previous research (Zeng et al., 2016), this study showed a relationship between higher levels of Streptococcaceae and *Streptococcus* and lower adherence to the MD. A relationship between high levels of these bacteria was also found with higher BMI. A high-fat diet, which contrasts the dietary pattern of the MD, greatly increased the relative abundance of Streptococcaceae, especially bacteria belonging to the genus *Streptococcus.* A long-term high-fat diet may produce not only an increase in inflammatory status but also an increase in Lachnospiraceae and Streptococcaceae bacteria in C57BL/6 mice (Zeng et al., 2016). It is well established that diet influences our health status. But until now has been unclear the contribution of microbiota to this effect. Curiously, in our study we could observed the same associations between some bacterial groups, for weight status as well as for MD adherence. This indicates that diet, health status, and microbiota may be interrelated.

### Dietary Nutrient Intakes

Animal protein, saturated fats, and simple sugars are nutritional components commonly found in the Western dietary pattern. This study found that high consumption of these nutrients was associated with a decrease in microbial richness and diversity. These results are in agreement with previous findings (Graf et al., 2015). There is increasing evidence suggesting that the overuse of antibiotics, clinical practices, and the type of diet usually associated with modern lifestyles promote changes in the human gut microbiota. This seriously affects microbial diversity (De Filippo et al., 2010; Yatsunenko et al., 2012; Segata, 2015) the depletion of which is associated with an increased risk of chronic diseases. Consumption of dietary fiber is very low in industrialized societies. Low fiber intake is likely to lead not only to a reduction in gut microbial composition and diversity but also to a reduction in the production of SCFA, which has important beneficial properties for human health (Jew et al., 2009; Huda-Faujan et al., 2010; O’Keefe et al., 2015; Sonnenburg et al., 2016; Sonnenburg and Sonnenburg, 2014). In accordance with previous studies, this study shows associations between the nutritional components of traditional Mediterranean-style diets, certain groups of beneficial bacteria, and fermentable end products (Singh et al., 2017). Also in agreement with the literature, higher total SCFA concentrations were found in individuals with a higher MD score (Gutiérrez-Díaz et al., 2016). Similar correlation patterns were found for two bacterial groups, which have been characterized by their potential importance for human health and leanness. These two groups are *Oscillospira* and *Butyricimonas*. Here, negative correlations were found between these groups and energy intake, animal proteins, saturated fats, and refined carbohydrates. *Butyricimonas* is a butyric-acid-producing genus of the phylum *Bacteroidetes* (Konikoff and Gophna, 2016; Shin et al., 2016). *Oscillospira* has been related with health and leanness (Konikoff and Gophna, 2016). In addition, recent works showed that Africans tend to have a higher level of *Oscillospira*, which might be involved in the assimilation of non- digestible carbohydrates (Walker et al., 2011; Tims et al., 2013). The MD contains a large proportion of vegetal food compounds. In addition, at higher levels of vegetal food compounds intakes, such as vegetal proteins, polysaccharides and dietary fiber, there were significantly higher bifidobacterial counts and significant positive correlations with various groups of butyrate-producing bacteria within the fecal microbial composition, along with higher concentrations of total SCFA. Some studies report similar shifts in gut microbiota and metabolic profile in association with consumption of these nutritional components (Lopez-Legarrea et al., 2014; Gutiérrez-Díaz et al., 2016; Singh et al., 2017). Dietary proteins affect gut microbiota differently depending on the source (Singh et al., 2017). Two recent works reported that consumption of plant protein increased *Bifidobacterium* and *Lactobacillus* genera, decreased the pathogenic bacterial species of *Bacteroides* and *Clostridium* genera, and increased SCFAs (Meddah et al., 2001; Kim et al., 2014). Moreover, there are studies showing that a high animal protein/low carbohydrate diet reduced bacterial groups including *Roseburia* and *Eubacterium*, which are well known butyrate producers (Rivière et al., 2016). Correlations were found between vegetal proteins and other butyrate-producing bacteria, such as *Dorea* and *Coprococcus*. Vegetal proteins were also correlated with higher counts (qPCR) of *Bifidobacterium.* All of these bacteria also had relationships with higher amounts of SCFA. Carbohydrates, especially non-digestible carbohydrates such as fiber and resistant starch, have been described (Graf et al., 2015) as having the highest impact of all nutritional components on gut microbiota composition, diversity, and metabolic profile. They exert a prebiotic potential, which stimulates the activity and growth of some specific groups of bacteria, for example *Bifidobacterium* and lactic acid bacteria. When they reach the large intestine, they are fermented by microbes producing SCFAs, which play an important role in human health. The MD is characterized by high consumption of foods that are sources of prebiotics, such as pulses, vegetables, whole grains, and fruit. A strong positive correlation was found in this study between SCFAs levels and MD score, as previously reported (Gutiérrez-Díaz et al., 2016, 2017). Moreover, studies have proven not only that plant based diets are linked to an increase in *Bifidobacterium* and *Lactobacillus* spp. but also that they can stimulate the growth of other beneficial bacterial species, especially bacterial communities involved in butyrate and methane production (Lockyer and Nugent, 2017). It has been reported that *Eubacterium rectale, F. prausnitzii, Roseburia* spp*., Dorea* and *Coprococcus* consume plant-vegetables to produce the SCFA (Rajilić-Stojanović de Vos, 2014; Rivière et al., 2016). Most of these bacterial groups were also associated with better cardiometabolic profile (Moens et al., 2017). Although *Bifidobacteria* produce acetic and lactic acid as main fermentation end products, they are implicated in butyrate production as a result of cross-feeding interactions between bifidobacteria and butyrate producing bacteria. This could mean that the presence of one group could favor the colonization of other bacteria (Rivière et al., 2016). This is in line with findings from the current study, which showed that consumption of plant-based nutrients was associated with the presence of *Bifidobacterium* as well as with some butyrate producers.

### Study Limitations

Limitations of this study include sample size, which could have affected the statistical power of the study. Furthermore, the FFQ was chosen because is the most complete tool to estimate intakes over an extended period of time (Serra Majem et al., 2006). However, it may have introduced bias caused by errors in memory and perception of food proportion sizes. Additionally, the FFQ is dependent on regular eating habits. It is also important to take into account, that it is very difficult to evaluate the influence of a dietary pattern only by dietary intakes analysis. There is increasing evidence that suggest that healthy dietary patterns are usually associated with other healthy-related habits. Moreover, the impact of environmental factors, including aspects of lifestyle, such as exercise or chronic stress on microbiota are also not studied in this work. Despite these limitations and the fact that more work is needed, the findings of this study are useful as they may help to promote further research in the field of dietary strategies, microbiota, and health.

## Conclusion

In conclusion, the main findings of this study showed that specific nutritional components and dietary patterns influence gut microbiota composition, diversity and activity. Associations were found between certain bacterial groups, weight and MD adherence. This means that nutritional strategies may have an influence on physiology and health through the microbiota. This study highlights the relevance of dietary components in the activity and composition of the microbiome in non-pathological adult individuals. Therefore, it is necessary to increase knowledge of the diet–microbiota interaction in order to design dietary strategies aimed to modulate the microbiota and at the same time to reduce the risk of disease.

## Author Contributions

IG-M and MC both planned the experiments and wrote the manuscript, and both accepted the final version of the manuscript. IG-M, MS-R, and CA analyzed the data and criticized the manuscript. All authors reviewed the manuscript.

## Conflict of Interest Statement

The authors declare that the research was conducted in the absence of any commercial or financial relationships that could be construed as a potential conflict of interest.

## Footnotes

1 http://cgenome.net/calypso/

## Supplementary Material

FIGURE S1 Pearson correlations heatmap between the nutrients and the relative abundances of specific gut bacteria at genus level present healthy adults (*n* = 27). Each column represents each nutrient and each row represents one genus. ^∗^ represents significant *P* < 0.05 correlation. Red: positive association and blue: negative association.

TABLE S1 Significant correlations between the nutrients and the relative abundances of specific gut bacteria at genus level.

## Abbreviations

BMI: body mass index
HPLC: high performance liquid chromatography
MD: Mediterranean diet
SCFA: short chain fatty acids
