# Supplementary figures and images for: Shifts on Gut Microbiota Associated to Mediterranean Diet Adherence and Specific Dietary Intakes on General Adult Population
Source: Front Microbiol. 2018 May 7;9:890. doi: 10.3389/fmicb.2018.00890 (PMC5949328; doi:10.3389/fmicb.2018.00890)

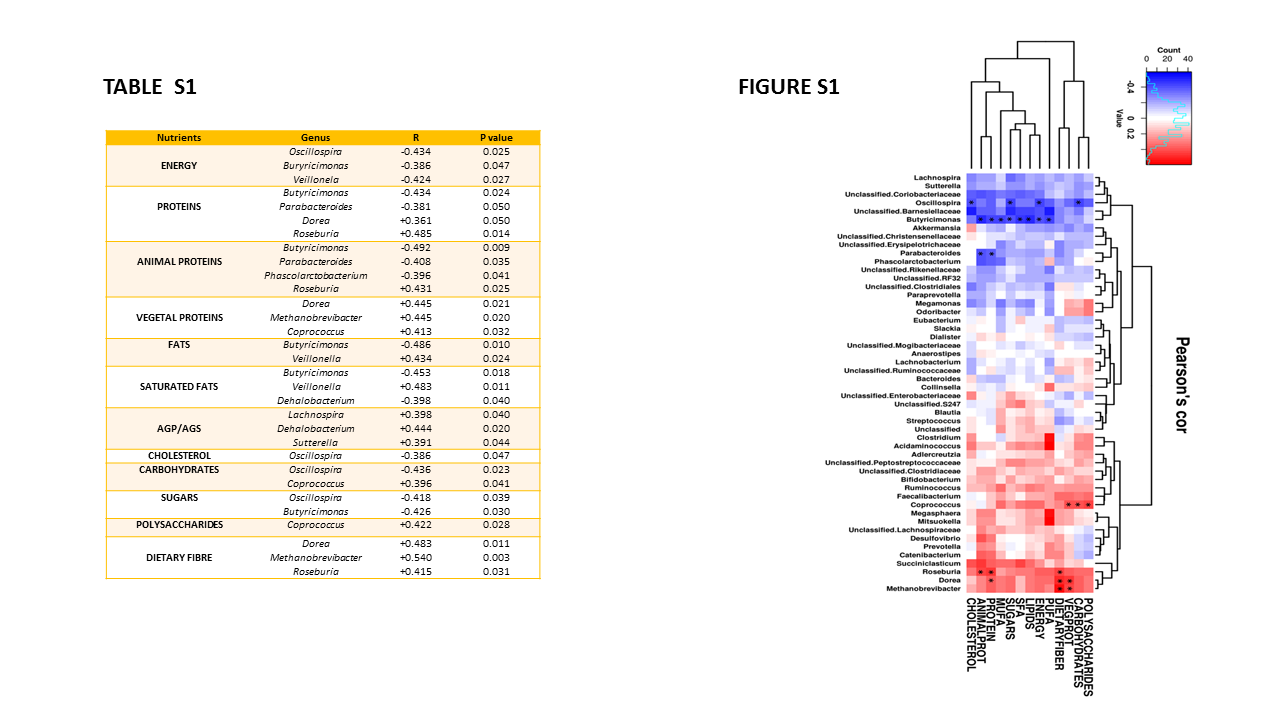

Supplement: FIGURE S1 — Pearson correlations heatmap between the nutrients and the relative abundances of specific gut bacteria at genus level present healthy adults (n = 27). Each column represents each nutrient and each row represents one genus. ∗ represents significant P < 0.05 correlation. Red: positive association and blue: negative association. [file Image_1.TIF]
